# Longitudinal high-risk sibling studies of autism spectrum disorder

**DOI:** 10.3402/mehd.v23i0.18983

**Published:** 2012-08-24

**Authors:** Terje Falck-Ytter

**Affiliations:** Center of Neurodevelopmental Disorders at Karolinska Institutet (KIND), Stockholm, Sweden

**Keywords:** Autism, sibling, recurrence rate, development, diagnosis, longitudinal design

Autism spectrum disorder (ASD) is a largely genetic (1, but also see 2) and highly prevalent (3) neurodevelopmental disorder. It is characterized by significant social impairments, and is associated with significant suffering and societal costs (4, 5). Currently, ASD cannot be diagnosed reliably before the age of 2 years. Although biomarkers for ASD are emerging (6), there are no biomarkers available that can prospectively predict diagnostic outcome at early ages.

The ASD risk in siblings of individuals with an ASD diagnosis may be as high as 20%, on average (7). The recurrence rate is much higher in boys than in girls, and higher in multiplex families compared to simplex families. In North America and the UK, emphasis is currently placed on prospective longitudinal studies of infant siblings of children with ASD in order to better understand the condition's early neurodevelopmental trajectories and to identify early biomarkers. Siblings, alongside typically developing low risk controls, are monitored during the first 2 years using both established clinical instruments (e.g. child observation, interview with parents) and novel methods (e.g. eye-tracking, brain imaging). This period is critical in order to discover novel (bio-)markers as well as map out the emergence of symptoms. Later, typically at 36 months, a complete diagnostic evaluation is performed. Improving the early detection of ASD is a pivotal aim of clinical services, as it is a precondition for early intervention, which may improve long-term outcomes for individuals with ASD.

Recent high-risk infant sibling studies have revealed a number of potential early markers, primarily in social communication, motor function, and attention domains, and mostly emerging around the younger sibling's first birthday (8). However, few markers have been identified before the age of 12 months, and the identified 12-month markers are not good predictors at an individual level.

Very recent evidence suggests that brain based biomarkers may be better early predictors of ASD than traditional measures of early ASD symptoms. In particular, one study found that the developmental trajectories of several major white matter fiber tracts were altered in high-risk siblings who later received an ASD diagnosis compared to high-risk siblings who did not receive a diagnosis at follow-up (9). This study suggests that early structural brain abnormalities may be important for understanding ASD, although a limitation of this study was the lack of neurotypical low-risk controls. Another recent study (10) used event related potentials (ERP), and found that those high risk siblings who later received a diagnosis showed different ERPs to direct gaze compared to high risk controls who did not receive a diagnosis later on (as well as compared to low risk controls). It is not unlikely that these functional and structural abnormalities are indeed accompanied by yet unidentified differences in attention and other overt behaviors.

To fully understand development, one must follow the individual longitudinally, and high risk infant sibling studies provide a basis for understanding complex developmental processes related to ASD. One important avenue for future ASD research is to map environmental risk factors and developmental processes during the first years of life. In combination with genetic information and the development of appropriate animal models, such studies will give rise to fundamental insight into the process by which an early alteration may have cascading developmental consequences, and ultimately lead to (perhaps years later) a formal clinical diagnosis.

In addition to describing these findings, in this talk I will also describe Early Autism Sweden (EASE; http://www.earlyautism.org), currently the only high risk sibling study in the Nordic Countries. EASE is a collaboration between the Center of Neurodevelopmental Disorders at Karolinska Institutet (KIND; http://www.ki.se/kind) and the Uppsala Babylab (http://www.babylab.se). This longitudinal and multi-method project is one of several nodes in a large European Innovative Medicines Initiative (IMI) network called EU-AIMS (http://www.eu-aims.eu/). A central goal of this network is to develop the infrastructure underpinning new treatments for autism.
